# Sapsucker Wells as a Keystone Nutritional Resource: Evaluating Methods for Detection of Secondary Sap Consumers

**DOI:** 10.1002/ece3.72277

**Published:** 2025-10-09

**Authors:** Rick Clawges, Shannon Blair, Jan Eitel, Leona K. Svancara, Lee Vierling, Kerri Vierling

**Affiliations:** ^1^ Department of Fish and Wildlife Sciences University of Idaho Moscow Idaho USA; ^2^ College of Natural Resources University of Idaho Moscow Idaho USA; ^3^ Department of Natural Resources and Society University of Idaho Moscow Idaho USA; ^4^ U.S. Geological Survey Northwest Climate Adaptation Science Center Corvallis Oregon USA

**Keywords:** biodiversity, camera trapping, direct observation, DNA metabarcoding, Picidae, species interactions

## Abstract

North American sapsuckers are considered double keystone species because they (1) excavate nest cavities that are used by other birds, small mammals, and invertebrates, and (2) create and maintain sap wells, a temporary nutritional resource available to a variety of secondary consumers. Most previous reports of secondary sap consumption relied on human observers and were based on either brief or incidental observations. However, modern technology can greatly enhance observational techniques and provide additional insights into the functional, community‐level importance of sap wells. We used visual surveys, camera traps, and environmental DNA (eDNA) to identify secondary consumers of sap from wells created by red‐naped ( *Sphyrapicus nuchalis* ) and Williamson's ( *S. thyroideus* ) sapsuckers among three functional groups of shrubs and trees in south‐central Colorado: shrub willows (*Salix* spp.), Rocky Mountain maple ( *Acer glabrum* ), and conifer trees (Pinopsida). Camera traps and eDNA revealed additional sap‐well visitors not identified from direct observations. Camera traps were effective for detecting nocturnal sap‐well visitors such as small rodents as well as occasional diurnal visitors. Environmental DNA analyses corroborated findings from other methods and identified four additional taxa as possible sap consumers. The physiology of sap‐well visitors, such as the ability to taste and assimilate compounds within sap, may aid in determining consumption versus contact when evaluating the results of eDNA analyses. Total vertebrate taxa detected using all 3 methods included 17 bird taxa in 10 families within 3 orders and 8 mammal taxa in 6 families within 4 orders. Shrub‐willow sap wells attracted the most diverse vertebrate taxa (23), followed by Rocky Mountain maple (13) and conifer trees (10). Invertebrates in 13 families within 3 orders were observed feeding from sap wells during visual surveys. Because many secondary sap consumers perform ecological services such as pollination, seed dispersal, and pest control, the methods described here may aid in elucidating the importance of sap‐well creators in supporting biodiversity and ecosystem functioning.

## Graphical abstract

We used direct observations, camera traps, and environmental DNA (eDNA) to identify secondary consumers of sap from wells created by red‐naped ( *S. nuchalis* ) and Williamson's ( *S. thyroideus* ) sapsuckers among three groups of shrubs and trees in south‐central Colorado, USA. Camera traps were effective for detecting nocturnal sap‐well visitors such as small rodents, as well as occasional diurnal visitors. Environmental DNA analyses corroborated findings from other methods and identified additional taxa as possible sap consumers.

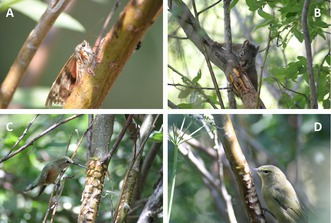

## 1 Introduction

Sap wells created by woodpeckers (Piciformes: Picidae) have long been known to provide nutrition to secondary consumers (Bolles 1891; Danforth 1938; Turcek 1954). Among woodpeckers, sapsuckers (*Sphyrapicus* spp.) are perhaps the best‐known group of sap‐well creators. Sapsuckers maintain well systems over a period of days or weeks on a few select trees and shrubs, attracting repeat visits by sap consumers, analogous to a backyard bird feeder (Foster and Tate 1966) or a temporary watering hole (Epaphras et al. 2007). Indeed, a wide variety of taxa have been recorded to visit sap wells created by sapsuckers (Foster and Tate 1966; Sutherland et al. 1982; Ehrlich and Daily 1988; Rissler et al. 1995). The diversity of secondary consumers supported by sap flows from wells has led some researchers to consider sapsuckers as keystone species because of their ability to drill sap wells as well as tree cavities, providing resources for numerous other species (Daily et al. 1993). Sapsucker wells are also considered one form of tree microhabitat (Larrieu et al. 2021; Asbeck et al. 2020). Byproducts of well drilling by sapsuckers may have uses for the broader ecological community beyond flowing sap. Resins, for example, may be produced in sap‐well drilling of conifer bark and are known to be used by some bees for nest construction, moisture regulation, chemical defense, and anti‐microbial properties (Chui et al. 2022).

Several different methods have been used to detect and identify sap‐well visitors. Foster and Tate (1966) conducted one of the earliest and most thorough studies of sap‐well visitors and their interactions, employing human observers, mist nets for bats, nets and fly paper for invertebrates, and still photography. All previous studies from 1966 to 2024 we reviewed that documented secondary sap‐well visitors relied fully or partially on human observers (Table A1). These studies ranged in scope from incidental observations obtained while documenting the activities of sap‐well creators (e.g., Kitching and Tozer 2010) to studies focused primarily on secondary foragers (e.g., Montellano et al. 2013). Three studies employed still and/or video cameras (Foster and Tate 1966; Ehrlich and Daily 1988; Martin et al. 2009), and three studies reporting invertebrate sap‐well visitation involved specimen collection for identification (Foster and Tate 1966; Genise et al. 1993; Rissler et al. 1995). Moreover, most studies have been performed during the day, while only two reports included surveys of nocturnal visitors (Foster and Tate 1966; Ehrlich and Daily 1988).

Direct human observation is still commonly used for wildlife monitoring, but this method may have disadvantages such as high cost of field labor, observer training requirements, and observer fatigue, as well as biases from survey timing and potential animal avoidance of observers (Zwerts et al. 2021). Currently, neither modern camera‐trapping technology nor environmental DNA (eDNA) has been employed to identify sap‐well visitors, yet these techniques have been successfully used for wildlife detection (including rare and elusive species) in terrestrial environments (Zwerts et al. 2021; Allen et al. 2023). Camera trapping allows for continuous sampling and can increase the detection potential of at‐risk species (Dinata et al. 2008; Huarcaya et al. 2020). Environmental DNA‐based approaches have been successfully used to sample a wide array of physical and biological media and may aid in exploring ecosystem‐level processes (Bohmann et al. 2014). Moreover, eDNA can be used as a non‐invasive complement to camera trapping and may be especially useful in detecting wildlife like small mammals that may elude detection on camera traps (Leempoel et al. 2020). The overall objective of this study was to determine how camera trapping and eDNA metabarcoding techniques may confirm and extend traditional observation surveys to identify visitors at sap wells created by sapsuckers. Our specific objectives were to (1) determine if eDNA from sap sources could be successfully extracted, amplified, and sequenced; (2) examine differences in the detection of sap‐well visitors by survey method and vegetation type; and (3) suggest modifications to improve the identification of sap consumers.

## 2 Materials and Methods

### 2.1 Study Design

#### 2.1.1 Study Area

**FIGURE 1:**
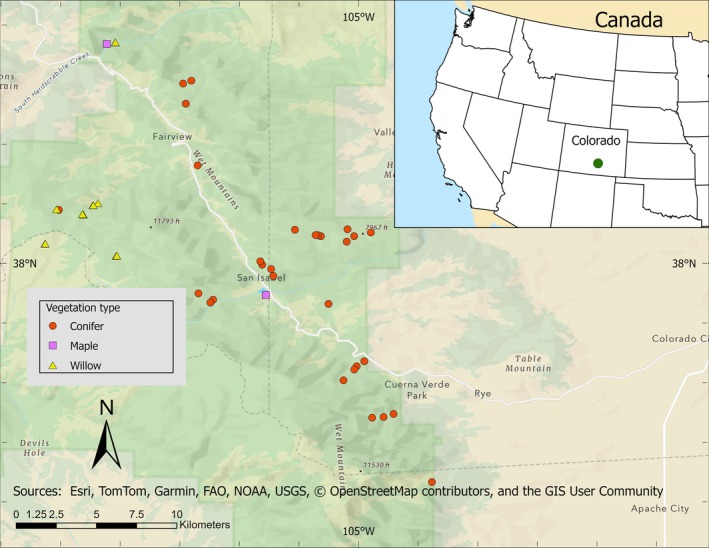
Map of sites in south‐central Colorado, USA, where the study was conducted.

We conducted this study from 27 March through 7 October 2022 in the Wet Mountains of south‐central Colorado, USA, in Custer, Pueblo, and Huerfano Counties (approximately 38°00′ N and 105°00′ W) (Figure 1). Land is primarily owned and managed by the United States Forest Service (USFS) with some private and state holdings. The USFS manages its holdings for recreation, wildlife, grazing, and timber production. Forested areas are largely mixed‐conifer, with Ponderosa pine ( *Pinus ponderosa* ) dominating lower elevations; Douglas fir ( *Pseudotsuga menziesii* ), white fir ( *Abies concolor* ), quaking aspen ( *Populus tremuloides* ), Englemann spruce ( *Picea engelmannii* ), and subalpine fir ( *Abies lasiocarpa* ) occur primarily at higher elevation areas with cooler north and east aspects. Willows (*Salix* spp.) may be abundant along streams and in subalpine valleys (Powell 2013).

#### 2.1.2 Sap‐Well Creators

**FIGURE 2:**
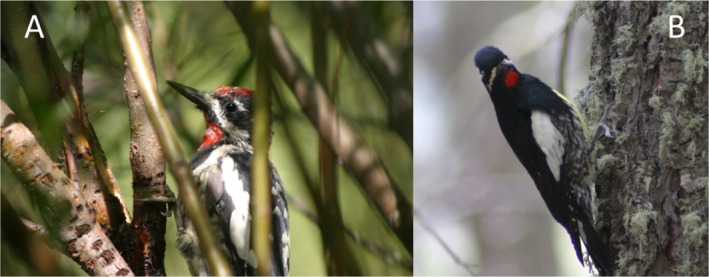
Two sapsucker species breed in south‐central Colorado, USA. The red‐naped sapsucker ( *Sphyrapicus nuchalis* ) (Panel A) drills sap wells in both conifer and broadleaf trees and shrubs (willow sap wells shown), while Williamson's sapsucker ( *S. thyroideus* ) (Panel B) drills sap wells exclusively in conifers. Photos by RC.

We focused our research on red‐naped ( *Sphyrapicus nuchalis* ) and Williamson's ( *S. thyroideus* ) sapsuckers (Figure 2) because both species have wide distributions and are known to create sap wells in trees and shrubs in mountainous areas of the Rocky Mountains in western North America. In Colorado, both sapsucker species are latitudinal migrants, arriving on breeding grounds within the state in late winter/early spring and departing in late summer/early autumn (Colorado Bird Atlas Partnership 2016). Red‐naped and Williamson's sapsuckers are known to use the same areas and occasionally the same trees for nesting from one breeding season to another, as well as foraging areas and sap‐well trees (Walters et al. 2020; Gyug et al. 2023). The red‐naped sapsucker is mostly associated with deciduous woody vegetation for sap extraction but will use conifer sap, particularly during colder parts of the year, because of almost year‐round flow and accessibility (Walters et al. 2020). Conversely, the Williamson's sapsucker feeds extensively on conifer sap and phloem fibers (Crockett 1975) but has not been reported to drill wells in deciduous sources (Gyug et al. 2023). Both red‐naped and Williamson's sapsuckers frequently excavate nest cavities in quaking aspen (Walters et al. 2020; Gyug et al. 2023). For the red‐naped sapsucker, quaking aspen is the most frequently selected nest tree in many parts of its range and is also used for sap feeding (Walters et al. 2020). Quaking aspen is declining in some parts of its North American range (Strand et al. 2009), with associated population declines in several aspen‐dependent woodpecker species, including red‐naped and Williamson's sapsuckers (Martin 2014). Global populations of both sapsucker species are considered Least Concern on the IUCN Red List (BirdLife International 2018, 2024). However, Williamson's sapsucker is listed as endangered in Canada because of habitat loss and small population size (COSEWIC 2017), and the U.S. Fish and Wildlife Service (USFWS) lists the breeding Rocky Mountains population in its Birds of Conservation Concern (USFWS 2021). Furthermore, the Williamson's sapsucker is considered an indicator of change in intensively managed forests of western North America (Conway and Martin 1993).

#### 2.1.3 Site Selection and Sap Resources

We conducted reconnaissance surveys in 2020 and 2021 by visually searching and listening for red‐naped and Williamson's sapsuckers to locate breeding and foraging areas within the study area. In 2022, we used call‐and‐drum playbacks (modified from Gyug et al. 2007) in known breeding and foraging areas as well as additional areas to locate and follow both species of sapsuckers to trees and shrubs used for sap feeding. Sap‐well visitors also aided in our discovery of flowing sap wells created by red‐naped sapsuckers within willow thickets. We located several well systems by following hummingbirds and butterflies, which frequent these well systems for sap consumption. We identified three key vegetation types used as sap sources by our focal species during the breeding season in the study area: Rocky Mountain maple ( *Acer glabrum* ), shrub willows ( *Salix brachycarpa* , *S. drummondiana* , *S. exigua* , and *S. monticola* ), and conifer trees (Pinopsida). Focusing our efforts on these vegetation types allowed us to sample sap from resources that differ in concentrations of compounds that may affect consumption by sap foragers. Sugar, for example, is believed to be an important energy source for sapsuckers and other sap feeders (Tate 1973; Miller and Nero 1983), and concentrations in sap can vary among plant species, vascular tissue used in transport (xylem or phloem), and season of the year (Fisher 1983; Jensen et al. 2016; Sperling et al. 2017). In addition to sugars, plant sap may also contain amino acids, proteins, minerals, and other compounds (van Bel 2003) that may act as PCR (polymerase chain reaction) inhibitors (Schrader et al. 2012; Soares et al. 2014). Sampling sap from a variety of vegetation types also allowed us to evaluate the effectiveness of eDNA methods to detect sap feeders among different plant sources.

The selection of sap sources used for feeding by sapsuckers varied over the breeding season. Red‐naped sapsuckers were observed using Rocky Mountain maple only in early spring before leaf out (9 April–6 May) when daytime temperatures typically exceed freezing, coinciding with increased sap flow and sugar concentrations within xylem (Milburn and O'Malley 1984; Gregory and Wargo 1986). Thus, the period for visual surveys, camera trapping, and collection of eDNA samples at maple sap wells was relatively brief (Table A2). Observations of sap‐well drilling in willows by red‐naped sapsuckers (30 June–20 September) and conifers by both sapsucker species (19 April–23 September) occurred over a longer period that allowed for a greater number of observer‐based surveys. Rocky Mountain maple and shrub willow typically occur as clumps < 4 m tall in the study area, which facilitated access for observation, camera placement, and eDNA sampling. Collection of camera‐trap data and eDNA samples at conifer sites was limited to fewer sites than willow because of physical access. We located four conifer trees with sap wells low enough above ground for camera placement and collection of eDNA samples. All of our study sites were located within the San Isabel National Forest and ranged in elevation from approximately 2600 to 3400 m in the montane and subalpine forested zones.

### 2.2 Survey Methods

#### 2.2.1 Direct Observation

We conducted 20‐min diurnal visual surveys for sap‐well feeders in three focal vegetation types from ground‐based observation points near used resources. Surveys were initiated 5 min after arrival at each site to allow potential sap‐well visitors to resettle after observer entry to the area. The same observer conducted all surveys between 09:30 and 16:30 h. Surveys near maple and within or adjacent to willow thickets were typically conducted at 5 m from a tree or shrub with active sap wells. Sap wells at these sites were approximately 1 m or less from the ground, so observation distances were less than 5 m in a few instances where ground‐level vegetation obstructed the view at 5 m. Sap wells in conifer trees ranged from ~1 m to over 10 m above ground and were viewed at ~10 m distance from the tree base using binoculars. After completing each 20‐min survey, we conducted a 5‐min close‐up survey of sap wells accessible from ground level using visual observations complemented with a hand lens to better examine invertebrate sap consumers. We photographed and/or collected specimens of select individual invertebrates for later identification if we could not identify them on site. Viewing distances employed in our study were similar in range (3–5 m) to those previously reported (Foster and Tate 1966; Ehrlich and Daily 1988; Williams 1990).

#### 2.2.2 Camera Trapping

We deployed camera traps equipped with automatic day/night motion‐detection sensors and infrared no‐glow LEDs (6 manufactured by Apeman, Shenzhen, China, and 2 manufactured by Stealth Cam LLC, Texas, USA) to record visitation of sap wells by sapsuckers and secondary foragers. We used a minimum time of 1 min between images for all camera traps and programmed cameras to capture images of sap‐well visitors continuously (day and night). We checked camera traps approximately weekly to verify functioning and re‐position cameras to optimize image capture of active sap wells when necessary. We also replaced camera batteries (4–8, depending on the model) and memory cards (16–32 GB) as needed during the weekly visit. All images were viewed by a single analyst who identified sap consumers within each image to the lowest taxonomic level possible, given the distance and viewing conditions (available light and position of sap‐well visitor).

Prior to camera‐trap deployment, we performed test placements of cameras in maple and willow sap‐well systems at distances varying from 0.5 to 2 m to assess the effectiveness of image capture and identification potential for sap‐well visitors of varying sizes. Camera traps were placed ~1 m from sap wells in maple and willow systems to allow optimal detection and identification of visitors. At this distance, mammalian sap‐well visitors as small as mice and chipmunks triggered image capture and could be reliably identified at the family or finer level. Sap‐well systems drilled in conifer trees were typically larger (> 1 m in some instances), so cameras were placed 2–3 m away for a broader view; camera traps were only used at sap wells < 3 m from the ground on conifer trees because of the difficulty in mounting cameras using posts or adjacent trees in terrain that was often rocky and sloped.

#### 2.2.3 Environmental DNA

Environmental DNA samples were obtained by rolling a 16‐mm long, 4.8‐mm diameter foam‐tipped swab (#25‐1506 1PF 100 Puritan Medical Products Company) across flowing sap wells, residues below wells, and dry areas < 1 cm above the highest active sap well to capture DNA for climbing or perching sap‐well visitors. One sterile swab was used per sample, and the foam tip of each new swab was dipped in a tube of phosphate‐buffered saline (PBS) before eDNA collection. After sampling, the foam tip from each swab was placed in a separate 2.0‐mL leak‐proof screw‐top microcentrifuge tube filled with 0.7‐mL Queen's lysis buffer, allowing for 100% submergence of the foam swab tip in the buffer solution. A new pair of clean vinyl gloves was used in the collection of each sample, which was stored at room temperature until extraction.

Early attempts to use an invertebrate‐specific primer set were not successful, and the troubleshooting necessary to potentially produce usable data was beyond the scope and limited resources available for the study. However, our early attempts using a vertebrate primer set were successful. Given that we had both direct observations and camera‐trap data for vertebrates but lacked invertebrate camera‐trap data to compare to the other methods, we focused our efforts on vertebrates for the eDNA assessment. The methods and results using eDNA techniques presented in this paper are therefore limited to potential vertebrate sap consumers.

Molecular methodologies, bioinformatic processing, and vertebrate primers employed in this study were similar to those used by Paprocki et al. (2024). Briefly, we performed a two‐step library preparation. In the first round of PCR, we used the 12S‐V5 primers designed by Riaz et al. (2011) with custom sequencing overhangs. The second round of PCR added i5 and i7 indexes and barcodes described in Campbell et al. (2015) to each sample. The resulting library was cleaned and size‐selected using Ampure XP beads and visualized on a BioAnalyzer before sequencing. Sequencing was carried out using approximately 20% of an Illumina Novaseq 6000 S4 lane. All eDNA samples were run in duplicate, and results were de‐replicated after taxonomic assignment. Positive controls (DNA from species not known to consume sap) and negative (no‐template) controls were included in duplicate. The two positive control species we added were bighorn sheep ( *Ovis canadensis* ) and mountain lion ( *Puma concolor* ).

A custom local reference database was built using all potentially occurring taxa. Potential vertebrate taxa were identified using known distribution maps. We also included common contaminants (humans and domestic animals) and contaminants that may be specific to the lab in our local database to more easily identify off‐target sequences. We queried the National Center for Biotechnology Information (NCBI) Genbank for 12S V5 sequences of each potential vertebrate taxon on or about 1 August 2023 and formatted results as a Basic Local Alignment Search Tool (BLAST) database using a Python script we developed (https://github.com/sckieran/ediet_pipeline).

Next, we applied bioinformatic processing methods detailed in Paprocki et al. (2024). Briefly, sequences were trimmed of adapter sequences and filtered for quality using cutadapt v. 4.4 (M. Martin 2011), then forward and reverse reads were merged using PEAR (Zhang et al. 2014). Sequences were then collapsed into exact sequence variants (ESVs) using FASTX‐collapser (Hannon 2010), with singletons removed at this step. Finally, ESVs were filtered within each sample at 0.1% relative read abundance (RRA) to reduce noise (average read count cut‐off = 300 reads). We retained any ESV with > 500 reads regardless of RRA to avoid over‐filtering samples with unusually high read counts. We then queried our local reference database to create a list of vertebrate taxa detected using eDNA metabarcoding techniques. Because our local reference database may not have captured all species present in the study area, we also performed a remote search of the entire NCBI Genbank database for all unique DNA sequences that did not have a hit in the local reference database. Individual sequences were submitted for identification using BLAST (https://blast.ncbi.nlm.nih.gov/Blast.cgi) and assigned to the lowest taxonomic level represented by a single taxon among all top‐matching sequences with equal percent identity and alignment scores (≥ 97%). For example, if a sequence matched equally well to *Microtus longicaudus* and *Microtus pennsylvanicus* , the sequence would be assigned to *Microtus* sp. All unique taxa identified were examined. We retained avian and mammal taxa that are known to, or could potentially occur in the study area.

### 2.3 Sap Consumption

**FIGURE 3:**
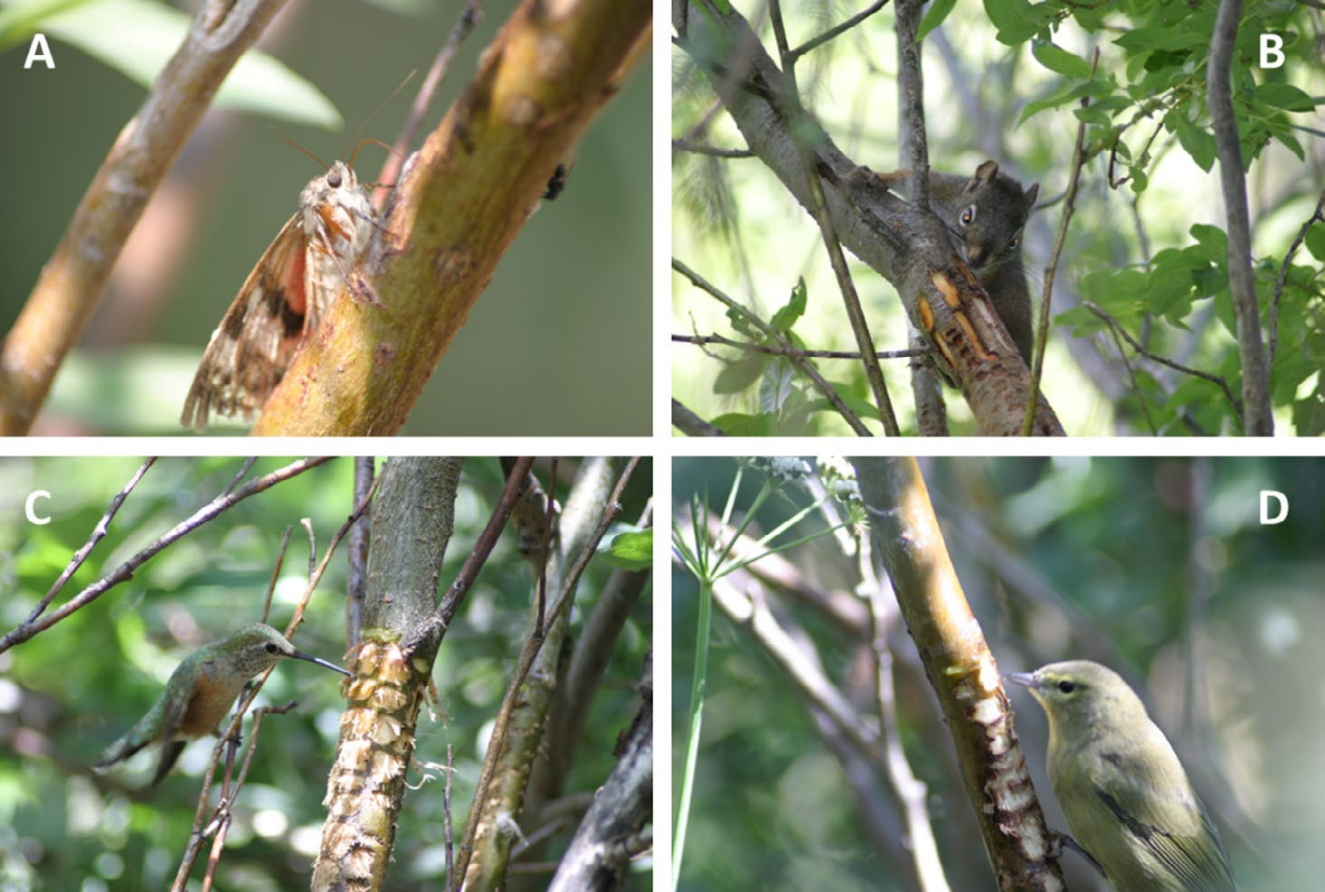
Common consumers of sap from sapsucker wells observed in south‐central Colorado, USA. (A) Underwing moth (*Catocala* sp.), (B) Red squirrel ( *Tamiasciurus hudsonicus* ), (C) broad‐tailed hummingbird ( *Selasphorus platycercus* ), and (D) orange‐crowned warbler ( *Leiothlypis celata* ). Photos by RC.

Sap wells are known to attract sap consumers and predators such as flycatchers and bats that feed on sap‐feeding arthropods (Foster and Tate 1966). In this study, however, we focused on direct consumers of sap. For visual surveys and examination of camera‐trap images, we considered taxa to be consumers if we observed mouthparts (e.g., tongue, beak, or proboscis) making contact with sap or sap residues (Figure 3). We report eDNA detections from sap‐well samples for all taxa known to occur in the study area as evidence of possible sap consumption and discuss methods that may be used to determine sap consumption versus incidental contact.

### 2.4 Data Analyses and Map

Data are available in Clawges et al. (2025). We used R version 4.4.1 (R Core Team 2024) to generate descriptive statistics, packages ggplot2 (Wickham 2016) and ggsci (Xiao 2024) to create bar charts, and ArcGIS Pro 3.3.0 (ESRI 2024) to create the study map.

## 3 Results

Vertebrate DNA was successfully extracted and amplified from sap samples taken from wells in all three of our focal vegetation types (Rocky Mountain maple, shrub willow, and conifer trees). DNAs from humans ( *Homo sapiens* ) and domestic animals (e.g., *Felis catus* ) were likely introduced during sample collection and were removed as potential sap feeders.

**FIGURE 4:**
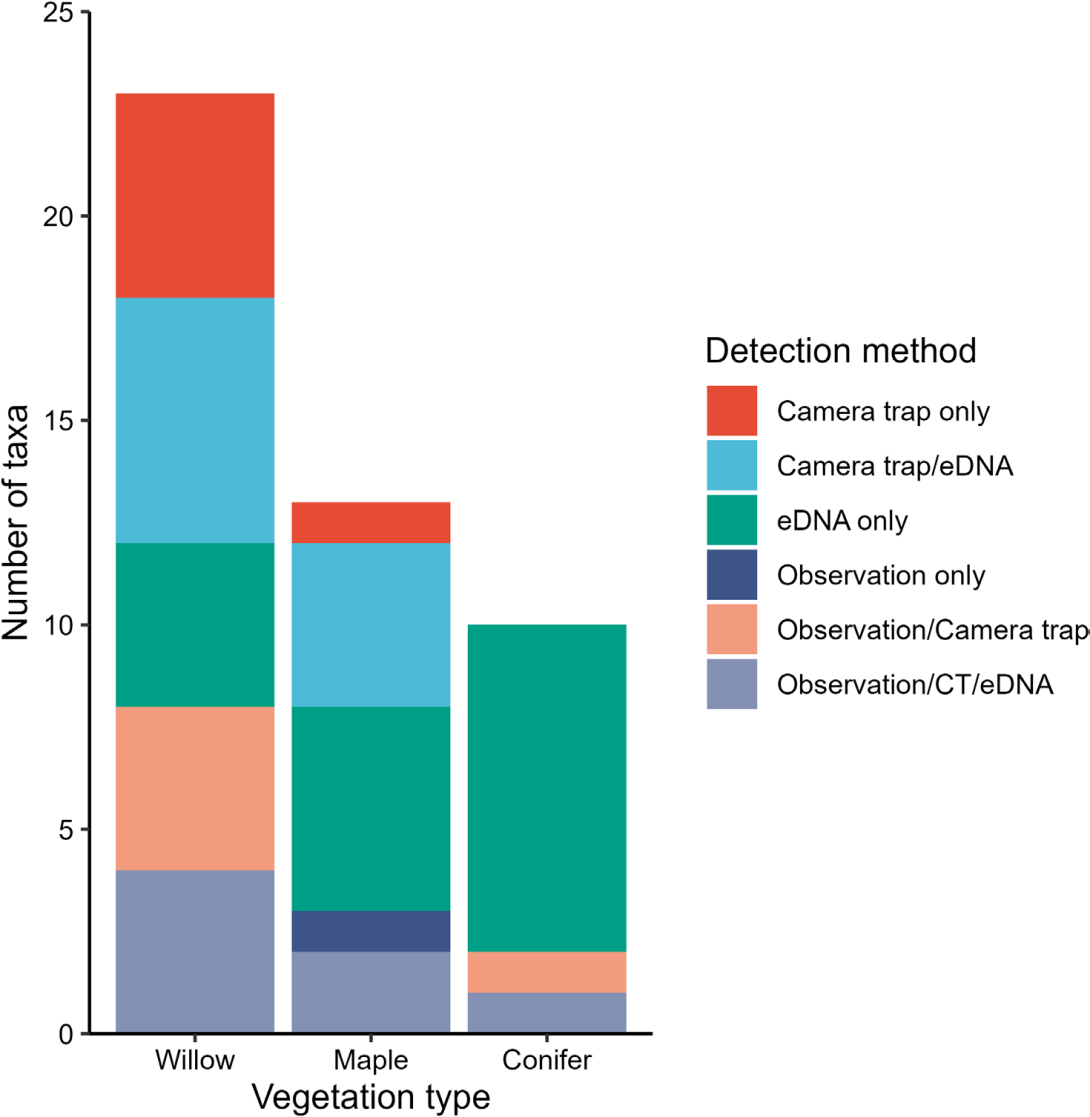
Number of total vertebrate taxa detected visiting sapsucker wells in south‐central Colorado, USA by detection method and vegetation type. CT, camera trap; eDNA, environmental DNA.

**FIGURE 5:**
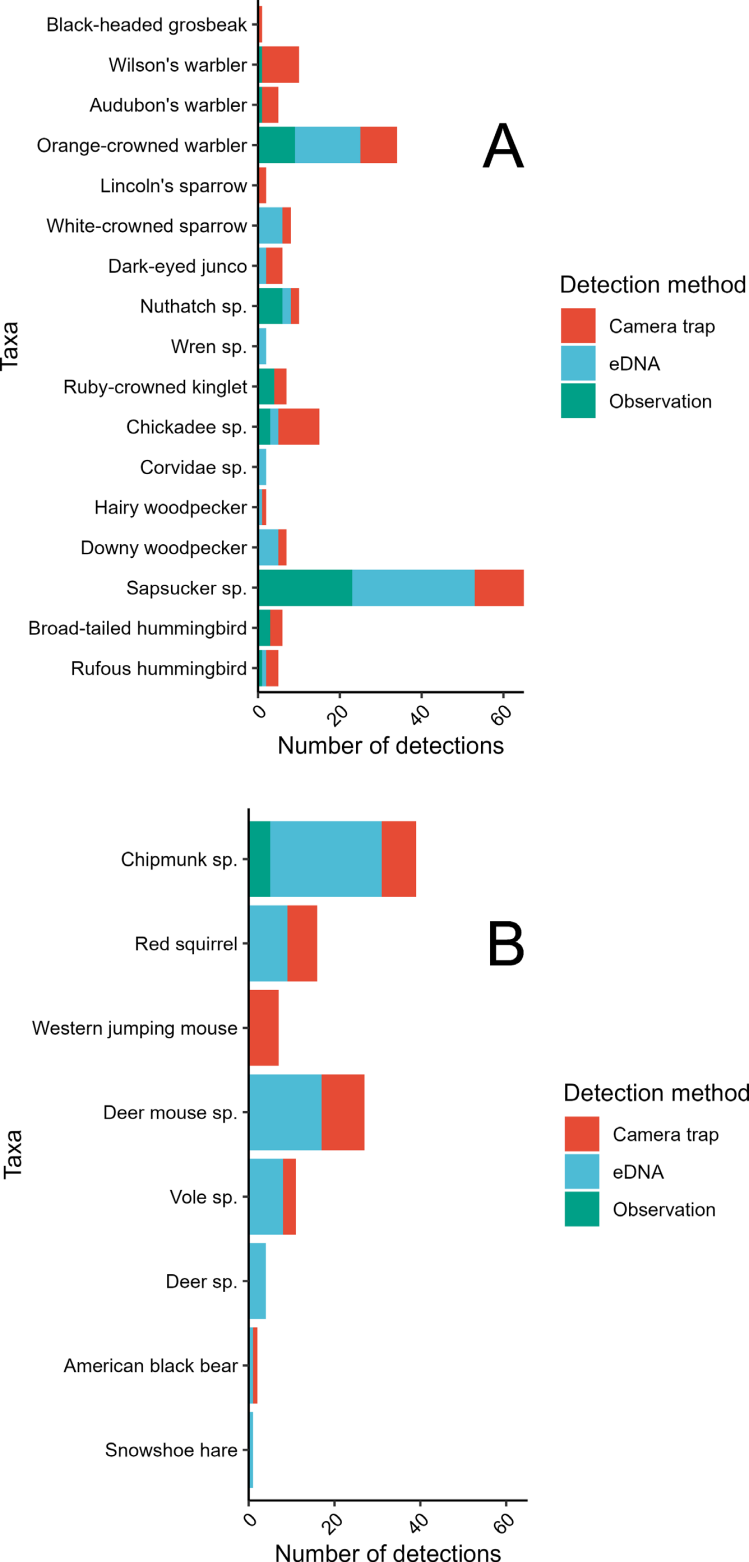
Number of detections of vertebrate taxa visiting sapsucker wells in south‐central Colorado, USA by method. Detections were summed for all vegetation types and represent the number of observation surveys, camera‐trap sites, and environmental DNA (eDNA) samples with detection of each taxon. Birds are shown in Panel (A) and mammals are shown in Panel (B).

**FIGURE 6:**
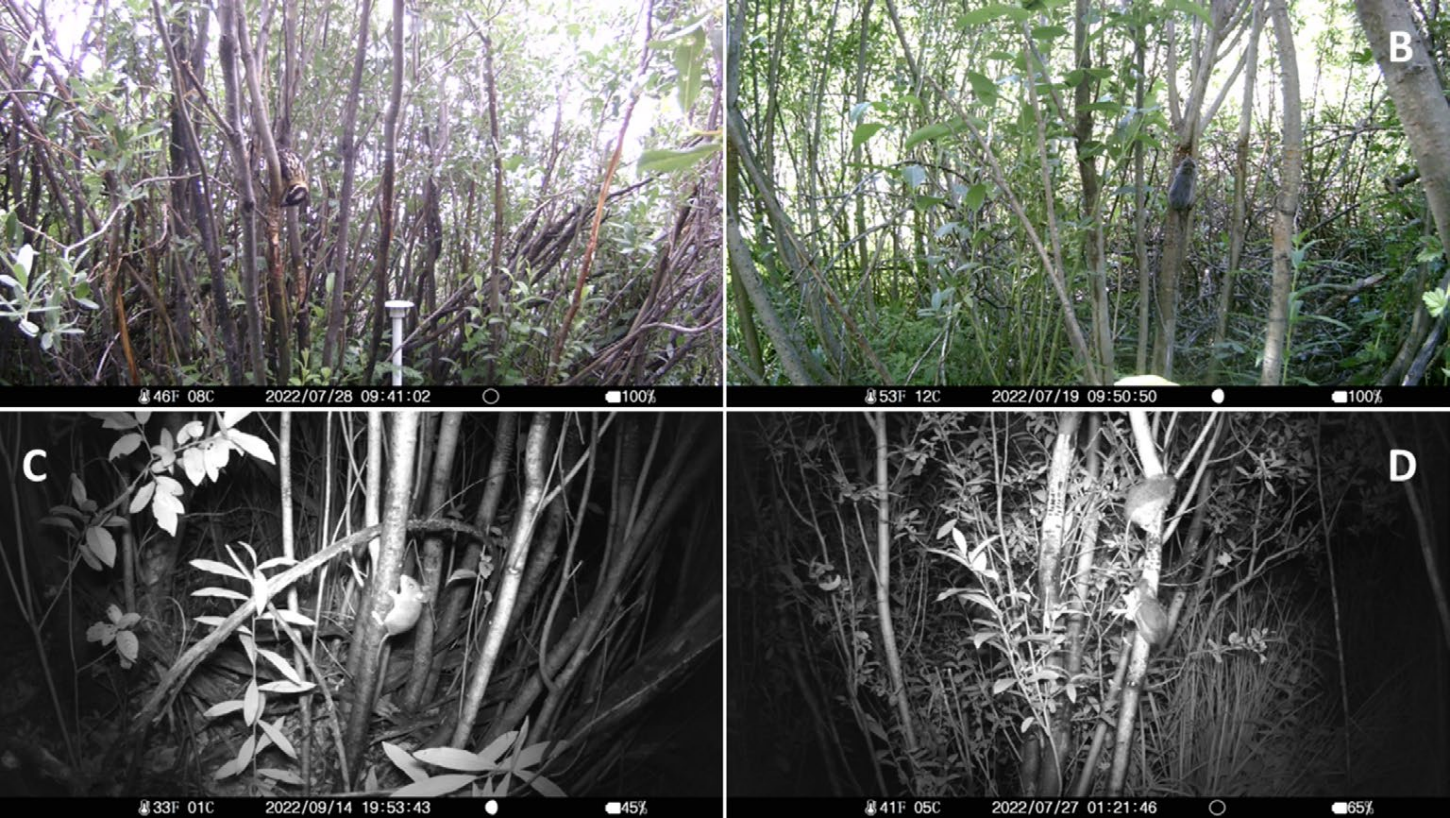
Sap‐well visitors detected with camera traps that were not detected during observation surveys: (A) black‐headed grosbeak ( *Pheucticus melanocephalus* ), (B) vole sp., (C) mouse sp., (D) western jumping mice ( *Zapus princeps* ).

Vertebrate taxa detected visiting sap wells in the three key vegetation types using all methods (observation surveys, camera trapping, and eDNA) included 17 bird taxa in 10 families within 3 orders and 8 mammal taxa in 6 families within 4 orders (Table 1, Table A3). Detections varied by method and taxa (Figures 4 and 5, Table 1). Camera traps captured the highest taxa richness (15 birds, 6 mammals), followed by eDNA (11 birds, 7 mammals) and observation surveys (9 birds, 1 mammal). While birds were commonly detected using all 3 methods, camera trapping and eDNA performed better than direct observations for the detection of mammals. While direct observation added only one new vertebrate species, pygmy nuthatch ( *Sitta pygmaea* ), to the list of sap‐well foragers detected during this study, camera‐trap images revealed several, including black‐headed grosbeak ( *Pheucticus melanocephalus* ) and voles (Figure 6) that have not been reported previously in the literature as sap‐well foragers. Taxonomic resolution differed by method as well. We were able to discern species for almost all vertebrates using direct observation, except on a few occasions where a sap feeder visited briefly and was obscured by vegetation. In contrast, vertebrates in some images were difficult to distinguish at the species level and were grouped to the genus level (*Poecile*, for example) or higher‐level animal group (e.g., vole sp.).

**TABLE 1:** Vertebrate taxa detected using sap wells drilled in three vegetation types by detection method.

Common name	Observations	Camera traps	Environmental DNA
	Number of surveys	Number of sites	Number of samples
Birds			
Rufous hummingbird^a^	1W	3W	1W
Broad‐tailed hummingbird	3W	3W	
Sapsucker sp.^b^	16W, 7C	10W, 1M, 1C	22W, 2M, 6C
Downy woodpecker		1W, 1M	5W
Hairy woodpecker		1M	1M
Corvidae sp.			2M
Chickadee sp.	3M	8W, 2M	1M, 1C
Ruby‐crowned kinglet	3W, 1M	3W	
Wren sp.			1W, 1M
Nuthatch sp.^b^	4M, 2C	1M, 1C	1W, 1M
Dark‐eyed junco		4W	2W
White‐crowned sparrow		2W	6W
Lincoln's sparrow		2W	
Orange‐crowned warbler	9W	9W	12W, 1M, 3C
Audubon's warbler	1W	4W	
Wilson's warbler	1W	9W	
Black‐headed grosbeak		1W	
Mammals			
Snowshoe hare^a^			1W
American black bear		1W	1C
Deer sp.			1W, 1M, 2C
Vole sp.		3W	5W, 3C
Deer mouse sp.		9W, 1M	13W, 1M, 3C
Western jumping mouse		7W	
Red squirrel		6W, 1M	6W, 1M, 2C
Chipmunk sp.	5W	8W	18W, 2M, 6C

*Note:* Vegetation types—W (willow sp.), M (Rocky Mountain maple), C (conifer sp.).

^a^ Identified as a Species of Greatest Conservation need (Colorado Parks and Wildlife 2015).

^b^ Individual species identified using visual methods (see Table A3) but not using eDNA analyses.

We examined the number of samples with detections using eDNA metabarcoding to assess frequency for each taxon and compared eDNA detections to those from visual methods to assess the plausibility of sap consumption by taxa (Table 1, Figure 5). Metabarcoding detections were corroborated by results from visual surveys and camera trapping for 14 of the 25 taxa detected. Red‐naped sapsucker, orange‐crowned warbler ( *Leiothlypis celata* ), and chipmunks (*Tamias* sp.) had relatively high numbers of eDNA sample detections and were detected in a relatively large number of visual surveys and camera‐trap sites as sap consumers. Chickadees (*Poecile* sp.) and nuthatches (*Sitta* sp.) were also detected by all 3 methods but less frequently. Four taxa detected only from metabarcoding [corvid sp. (Corvidae), wren sp. (Troglodytidae), snowshoe hare ( *Lepus americanus* ), and deer sp. (*Odocoileus* sp.)] were not confirmed as sap consumers using visual methods.

**FIGURE 7:**
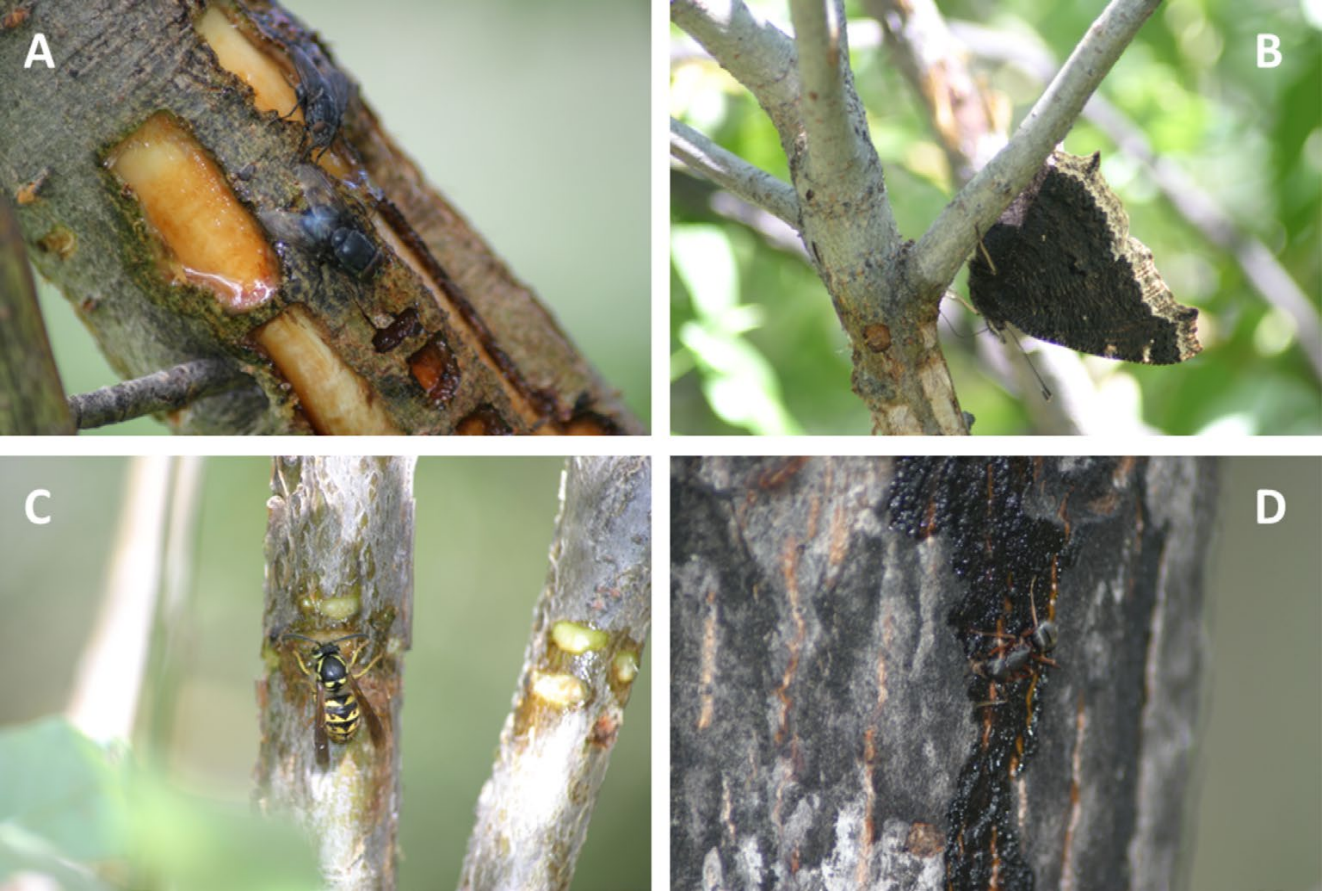
Invertebrates commonly observed feeding on sap and sap residue from wells drilled by red‐naped sapsuckers ( *Sphyrapicus nuchalis* ) in south‐central Colorado, USA. (A) Flies (Diptera), (B) mourning cloak butterfly ( *Nymphalis antiopa* ), (C) vespid wasp (*Vespula* sp.), (D) ant (Formicidae). Photos by RC.

Invertebrates in 13 families (9 Diptera, 2 Lepidoptera, 2 Hymenoptera) within 3 orders (Table 2) were observed feeding from sap wells in the three key vegetation types during visual surveys. Flying insects such as butterflies, dipterans, moths, and wasps (Figure 7) were commonly observed feeding on fresh sap from the highest active sap well, but also fed on sap residue from stems below non‐flowing sap wells. We observed 13 families of invertebrates during close‐up visual surveys and 6 using binoculars.

**TABLE 2:** Invertebrate taxa detected visiting sap wells in south‐central Colorado, USA.

Order	Family	Common name	Scientific name	Vegetation type		
				W	M	C
Lepidoptera	Nymphalidae	Comma/anglewing	*Polygonia* sp.	cl,s	cl,s	
		Milbert's tortoiseshell	*Aglais milberti*		s	
		Mourning cloak	*Nymphalis antiopa*	cl,s	cl,s	
	Erebidae	Grote's underwing moth	*Catocala grotiana*	cl		
Diptera	Psychodidae	Moth fly				cl
	Culicidae	Mosquito		cl		
	Syrphidae	Flower fly		cl		
	Tephritidae	Fruit fly		cl		
	Drosophilidae	Pomace fly		cl,s	cl,s	
	Muscidae	House fly		cl		
	Fanniidae	House fly			cl	
	Calliphoridae	Blow fly		cl,s	cl,s	
	Sarcophagidae	Flesh fly		cl,s	cl,s	
Hymenoptera	Vespidae	Yellowjacket	*Vespula* sp.	cl,s		
		Bald‐faced hornet	*Dolichovespula maculata*	cl,s		
	Formicidae	Ant		cl,s		cl

*Note:* Vegetation types – M, Rocky Mountain maple; W, willow species; C, conifer species. Values in vegetation type columns indicate survey type – cl, close‐up 5 min; s, standard 20 min binocular.

The number of sap‐well visitors detected varied by vegetation type. The number of vertebrate taxa detected from conifer sap wells (10) was similar to the number detected from maple (13), but only half of those detected from willow (23). Examining data from 20‐min visual surveys revealed that vertebrate sap consumers (not including sapsuckers) were observed in 24 of 43 (56%) surveys conducted at broadleaf shrubs (maple and willow combined) compared to 2 of 37 (5%) surveys conducted at conifer trees. Among the 13 invertebrate families detected from visual surveys, the number visiting conifer sap wells was low (2) compared to deciduous sources (5 for maple and 11 for willow).

## 4 Discussion

**FIGURE 8:**
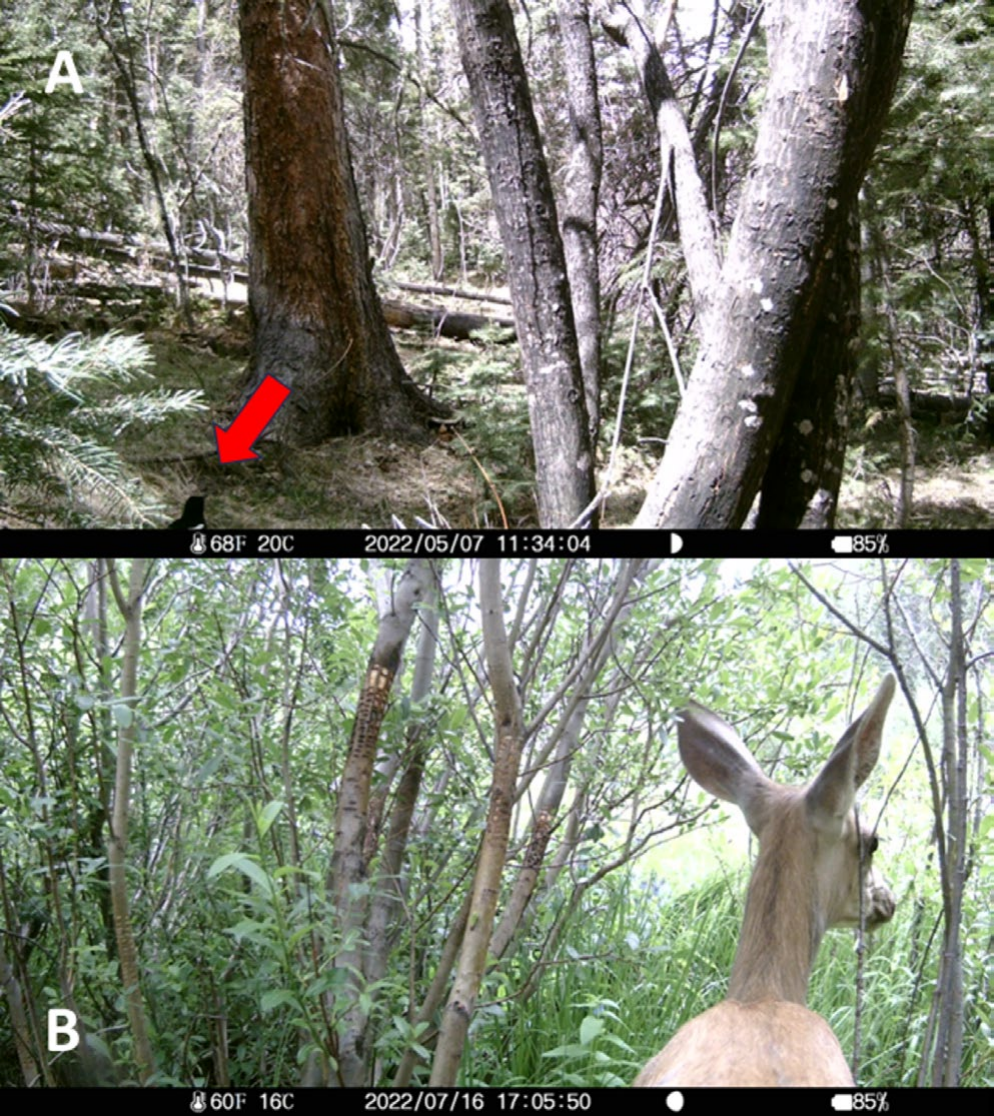
Examples of taxa detected from environmental DNA (eDNA) sampling that were not confirmed by visual methods to consume sap from sapsucker wells: Black‐billed magpie ( *Pica hudsonia* ), a member of the Corvidae family (Panel A), and mule deer ( *Odocoileus hemionus* ) (Panel B).

Camera trapping and eDNA sampling complemented direct observations to reveal a diverse community of wildlife attracted to sapsucker wells. Environmental DNA metabarcoding analyses detected vertebrates observed as sap‐well feeders in previous studies and identified several others as possible consumers. Corvids and deer were detected by camera traps (Figure 8), and their eDNA was detected in several sap samples, but neither taxon was confirmed as a sap consumer during the examination of images. The snowshoe hare is known to browse on willow leaves (Ellsworth and Reynolds 2006) and was detected only by eDNA metabarcoding at one willow site. Wrens were detected only by metabarcoding in our study; however, the northern house wren ( *Troglodytes aedon* ) has been reported by direct observation to feed at sapsucker wells (Crockett 1975). Detection of eDNA for some or all of these taxa may represent incidental contact with sap wells rather than sap consumption. Vertebrates are known to forage for invertebrates trapped in or attracted to sap (Foster and Tate 1966; Chapman et al. 1999). Also, brief appearances and activities of some vertebrates may not have been recorded by camera traps, given that we used a minimum time of 1 min between image captures.

Evaluation of sap consumption versus contact when examining results of eDNA analyses from this and future studies may benefit by considering the physiology of detected taxa. Sap consumption is likely driven, in part, by the ability of each forager to taste and assimilate compounds contained within plant sap, which is primarily composed of sucrose, with concentrations varying in xylem and phloem seasonally and among species (Sauter 1980; Miller and Nero 1983; Douglas 2006). Among birds, for example, sweetness perception is known among hummingbirds, songbirds, and woodpeckers (Baldwin et al. 2014; Toda et al. 2021; Cramer et al. 2022), groups that were detected as sap consumers in this study. Nectar‐feeding birds such as hummingbirds have been shown to have greater sucrose assimilation efficiencies than passerines (Schondube and Martinez del Rio 2004), while some specific passerines like the American robin ( *Turdus migratorius* ), which was observed foraging for invertebrates at several of our study sites but did not consume sap, show digestive intolerance to sucrose (Karasov and Levey 1990). In addition to sucrose, plant sap may also contain amino acids, proteins, minerals, and other compounds (van Bel 2003; Lohaus 2022) that likely influence sap intake and digestion.

Sap wells drilled by sapsuckers have been shown to provide an important nutritional resource for breeding and migrating hummingbirds in North America (Southwick and Southwick 1980; Sutherland et al. 1982). Both the breeding broad‐tailed hummingbird ( *Selasphorus platycercus* ) and migrant rufous hummingbird ( *S. rufus* ) were detected as sap consumers in our study area, but were infrequently detected compared to passerines with lower sucrose assimilation efficiencies. Hummingbird nests were located within 300 m of sapsucker wells in the study by Southwick and Southwick (1980). We did not attempt to locate the nests of sap‐well foraging birds. Other factors that may have influenced hummingbird presence or absence at sap wells in our study area include hummingbird migration timing, interspecific competition (Bolles 1891; Foster and Tate 1966; Sutherland et al. 1982; Ehrlich and Daily 1988), availability of other food resources (Sutherland et al. 1982), and additional site‐specific factors.

Sap wells found in the three focal vegetation types were generally not located in the same areas, and thus differences in taxa attracted to specific vegetation resources may be attributable in part to variations in elevation, terrain, dominant vegetation communities, and other factors. Similarly, sap wells could not be sampled at the same time in the three vegetation types, which may have influenced use potential for taxa such as migratory birds and insects that may not have been present during periods early and late in the sapsucker breeding season. Use of maple occurred in early spring when other food resources, such as invertebrates, were more limited, which may have added to its appeal to some sap consumers. The highest number of taxa were detected in shrub willow, possibly because willow was monitored more intensely and over a longer time period.

Sap consumers detected during this study are known to perform a variety of ecological services. Hummingbirds and some members of the orders Diptera, Hymenoptera, and Lepidoptera are valuable pollinators (Shepherd et al. 2003; Camfield et al. 2020). Small rodents disperse seeds, create burrows that are used by other species, and are prey items for other mammals and raptors. Rodents such as voles contribute not only to soil aeration but also nutrient fluxes (Wilske et al. 2015). Vespid wasps serve as prey for sapsuckers and other birds and are considered valuable as generalist predators of a wide range of pest insects (Donovan 2003). Other sap‐using invertebrates we detected function as scavengers (ants) and decomposers (dipterans) (Eaton and Kaufman 2007).

We acknowledge limitations to the analyses presented and offer modifications and improvements to be considered in future studies of sap‐well use. Our main research objective was to assess the utility of alternative methods to observer surveys. However, a more balanced number of observation surveys, camera hours, and eDNA samples would have allowed quantitative comparisons of sap‐well visitors among methods and vegetation types, but it was difficult to achieve given the variation in use of specific sap resources by sapsuckers for well creation. We focused on vertebrates and macroinvertebrates associated with sap wells. Larval insects and micro‐organisms such as yeast are known to occur in sap wells (Foster and Tate 1966; Kaczynski et al. 2014) and may benefit from sap‐well drillings. We experimented with video mode for camera traps early in the study, but found that memory cards filled up quickly, given the intensity of sap‐well visitation at many sites. However, video may improve taxa identification and aid in the confirmation of sap consumption. Using an image‐capture delay of less than 1 min for still images may improve detection of uncommon sap‐well visitors. The use of machine‐learning models for image classification and models of multi‐species interactions applied to camera‐trap data may aid in the identification of sap‐well visitors and analyses of interactions and behavioral patterns in feeding (Norouzzadeh et al. 2020; Nicvert et al. 2024).

We targeted the 12S gene using primers to amplify the DNA of vertebrates we thought were most likely to occur and feed from sap wells in our study area. Alternative genes and primer sets may be used to focus on specific taxa such as birds and mammals (Ushio et al. 2018; Hernández‐Canchola et al. 2021). Reference DNA databases for some taxa are at times incomplete or contain incorrectly labeled sequences (Leempoel et al. 2020; Ruedi et al. 2023); the addition of sequences and correction of errors in reference databases will likely provide more reliable species identifications in the future. Successful sequencing of invertebrate DNA from sap sources will likely reveal other consumers not determined from visual methods. DNA metabarcoding shows promise as a useful tool in the identification of invertebrates that may be advantageous over standard techniques that often require considerable effort and expense in sample collection, sorting, and laborious taxonomic assignments by experts (Yu et al. 2012; Morinière et al. 2016; Watts et al. 2019). DNA metabarcoding has been used in ecosystem analyses to examine invertebrates and other microorganisms associated with wildflowers (Thomsen and Sigsgaard 2019), honey (Utzeri et al. 2018), and fluids from pitcher plants (Bittleston et al. 2015). Our study reports the first use of DNA metabarcoding to identify sap‐well visitors from sap samples; additional studies in substrates such as sap can be used to determine the ability of eDNA methods to detect target taxa in novel media (Newton et al. 2025).

Sap wells have been shown to be a significant component in the diets of breeding and migrating hummingbirds (Southwick and Southwick 1980; Sutherland et al. 1982). Additional studies aimed at quantifying the energy budgets of sap‐well visitors and estimating the proportion of energy requirements that are met by sap consumption may further our understanding of the importance of these features as keystone structures (Tews et al. 2004). We detected two species of greatest conservation need (rufous hummingbird and snowshoe hare) identified in Colorado's State Wildlife Action Plan (Colorado Parks and Wildlife 2015) during this study. Continued research on sap‐well use may yield additional detections of at‐risk species and may be used to inform future conservation plans for sap foragers and associated plant resources. Furthermore, these methods could be used to document sap consumers associated with natural plant exudations, which are currently known to include unlikely taxa such as lizards and spiders (Edwards et al. 2021; Suzuki and Sano 2021). Sap flows made available by sapsuckers and other organisms may act as an alternative food source during periods when other resources, such as nectar, fruits, seeds, and invertebrates, are scarce (Montellano et al. 2013, 2019). In areas predicted to experience increased aridity from climate change where sap‐well creators are present, sap resources may play an increasingly important role for well creators and secondary consumers, though climate‐change effects on factors such as sap‐flow phenology, quantity, and quality (Woodruff 2014; Sperling et al. 2017) would need to be considered.

## Author Contributions

**Rick Clawges:** conceptualization (equal), data curation (lead), formal analysis (lead), investigation (lead), methodology (lead), visualization (lead), writing – original draft (lead), writing – review and editing (equal). **Shannon Blair:** conceptualization (supporting), data curation (supporting), formal analysis (supporting), investigation (supporting), methodology (supporting), software (lead), writing – original draft (supporting), writing – review and editing (equal). **Jan Eitel:** conceptualization (equal), writing – review and editing (equal). **Leona K. Svancara:** conceptualization (equal), writing – review and editing (equal). **Lee Vierling:** conceptualization (equal), resources (supporting), supervision (equal), writing – review and editing (equal). **Kerri Vierling:** conceptualization (equal), resources (supporting), supervision (equal), writing – review and editing (equal).

## Conflicts of Interest

The authors declare no conflicts of interest.

## Data Availability

Data are openly available in VERSO at https://doi.org/10.60841/000000267 (Clawges et al. 2025). The sequencing data that support the findings of this study are available in the National Center for Biotechnology Information SRA repository under BioProject accession number PRJNA1283213 (https://www.ncbi.nlm.nih.gov/bioproject/PRJNA1283213/).

## Appendix A

**TABLE A1:** Summary of studies reviewed that reported secondary visitation of sap wells created by vertebrates.

Sap‐well creator	Secondary visitor source	Secondary visitors	Detection methods	Observation hours^a^
Acorn woodpecker	Kattan and Murcia (1985)	B	O	78
White‐fronted woodpecker	Genise et al. (1993)	B, M, I	O, IS	NA
White‐fronted woodpecker	Blendinger (1999)	B	O	~7^a^
White‐fronted woodpecker	Macchi et al. (2011)	B	O	18^a^
White‐fronted woodpecker	Montellano et al. (2013)	B	O	~175^a^
White‐fronted woodpecker	Montellano et al. (2019)	B	O	NA
Williamson's sapsucker	Crockett (1975)	B	O	750
Yellow‐bellied sapsucker	Foster and Tate (1966)	B, M, I	O, MN, IS, SC	250
Yellow‐bellied sapsucker	Southwick and Southwick (1980)	B, M, I	O	NA
Yellow‐bellied sapsucker	Williams (1990)	I	O	18^a^
Yellow‐bellied sapsucker	Rissler et al. (1995)	I	O, IS	8^a^
Yellow‐bellied sapsucker	Kitching and Tozer (2010)	M	O	NA
Red‐naped sapsucker	Ehrlich and Daily (1988)	B, M, I	O, SC	54
Red‐breasted sapsucker	Sutherland et al. (1982)	B, M, I	O	NA
White‐headed woodpecker	Kozma (2010)	B	O	NA
Magellanic woodpecker	Schlatter and Vergara (2005)	B	O	~5^a^
Glossy flowerpiercer	Martin et al. (2009)	I	O, VC	~4
Yellow‐bellied glider	Chapman et al. (1999)	B	O	NA

*Note:* Secondary visitors – B (birds), M (mammals), I (invertebrates); detection methods – IS (invertebrate specimen), MN (mist net for bats), O (human observer), SC (still camera), VC (video camera).

^a^ Hours estimated using details provided in study description.

**TABLE A2:** Summary statistics for methods used to detect vertebrate sap‐well foragers in three vegetation types in south‐central Colorado, USA.

	Willow	Maple	Conifer
Observation surveys			
Date of first survey	21 Apr	27 Mar	16 Apr
Date first flowing sap well found	24 Jun	9 Apr	19 Apr
Observation survey date range	30 Jun–27 Sep	9 Apr–6 May	19 Apr–23 Sep
Number of observation survey sites (repeat surveys)	11 (7)	2 (2)	27 (7)
Morning surveys	16	7	11
Afternoon surveys	17	3	26
Total surveys	33	10	37
Mean number of surveys per site (SE)	3.0 ± 0.5	5.0 ± 2.0	1.4 ± 0.14
Camera trapping			
Number of camera‐trap sites	10	2	4
Camera‐trapping date range	24 Jun–7 Oct	9 Apr–16 May	24 Apr–23 Jun
Total camera‐trapping days	451	56	60
Mean camera‐trapping hours per site (SE)	45 ± 5.3	28 ± 9	15 ± 5.5
Environmental DNA (eDNA)			
Number of eDNA sample sites (repeat samples)	12 (8)	2 (0)	6 (0)
Number of eDNA samples collected	22	2	6
eDNA sampling date range	24 Jun–20 Sep	3–6 May	1 May–19 Aug
Mean number of eDNA samples per site (SE)	1.83 ± 0.21	1 ± 0	1 ± 0

Abbreviation: SE, standard error.

**TABLE A3:** Vertebrate taxa detected visiting sap wells drilled in three vegetation types in south‐central Colorado, USA.

Birds	Scientific name	Order	Family	Vegetation type		
				W	M	C
Rufous hummingbird^a^	*Selasphorus rufus*	Apodiformes	Trochilidae	X		
Broad‐tailed hummingbird	*Selasphorus platycercus*	Apodiformes	Trochilidae	X		
Williamson's sapsucker	*Sphyrapicus thyroideus*	Piciformes	Picidae			X
Red‐naped sapsucker	*Sphyrapicus nuchalis*	Piciformes	Picidae	X	X	
Downy woodpecker	*Dryobates pubescens*	Piciformes	Picidae	X	X	
Hairy woodpecker	*Leuconotopicus villosus*	Piciformes	Picidae		X	
Corvid sp.	*Corvidae* sp.	Passeriformes	Corvidae		X	
Chickadee sp.	*Poecile* sp.	Passeriformes	Paridae	X	X	X
Ruby‐crowned kinglet	*Corthylio calendula*	Passeriformes	Regulidae	X	X	
Wren sp.	*Troglodytidae* sp.	Passeriformes	Troglodytidae	X	X	
Red‐breasted nuthatch	*Sitta canadensis*	Passeriformes	Sittidae		X	X
White‐breasted nuthatch	*Sitta carolinensis*	Passeriformes	Sittidae		X	
Pygmy nuthatch	*Sitta pygmaea*	Passeriformes	Sittidae		X	X
Dark‐eyed junco	*Junco hyemalis*	Passeriformes	Passerellidae	X		
White‐crowned sparrow	*Zonotrichia leucophrys*	Passeriformes	Passerellidae	X		
Lincoln's sparrow	*Melospiza lincolnii*	Passeriformes	Passerellidae	X		
Orange‐crowned warbler	*Leiothlypis celata*	Passeriformes	Parulidae	X	X	X
Audubon's warbler	*Setophaga auduboni*	Passeriformes	Parulidae	X		
Wilson's warbler	*Cardellina pusilla*	Passeriformes	Parulidae	X		
Black‐headed grosbeak	*Pheucticus melanocephalus*	Passeriformes	Cardinalidae	X		

Mammals	Scientific name	Order	Family	Vegetation type		
				W	M	C
Snowshoe hare^a^	*Lepus americanus*	Lagomorpha	Leporidae	X		
American black bear	*Ursus americanus*	Carnivora	Ursidae	X		X
Deer sp.	*Odocoileus* sp.	Artiodactyla	Cervidae	X	X	X
Vole sp.	*Microtus/Myodes* sp.	Rodentia	Cricetidae	X		X
Deer mouse sp.	*Peromyscus* sp.	Rodentia	Cricetidae	X	X	X
Western jumping mouse	*Zapus princeps*	Rodentia	Dipodidae	X		
Red squirrel	*Tamiasciurus hudsonicus*	Rodentia	Sciuridae	X	X	X
Chipmunk sp.	*Tamias* sp.	Rodentia	Sciuridae	X	X	X

*Note:* Vegetation types – W (willow sp.), M (Rocky Mountain maple), C (conifer sp.).

^a^ Identified as a Species of Greatest Conservation Need (Colorado Parks and Wildlife 2015).
